# Supplementary material for: DMRforPairs: identifying Differentially Methylated Regions between unique samples using array based methylation profiles
Source: BMC Bioinformatics. 2014 May 15;15:141. doi: 10.1186/1471-2105-15-141 (PMC4046028; doi:10.1186/1471-2105-15-141)
Supplement: Additional file 2 — DMRforPairs output for the comparison of A431-MCF7 and NA17018-NA17105. Please start from the HTML files in each folder. Available via the BMC Bioinformatics website. [file 1471-2105-15-141-S2.zip › 1394847754114233_MOESM2_ESM/A431_MCF7/figures/3157.pdf]

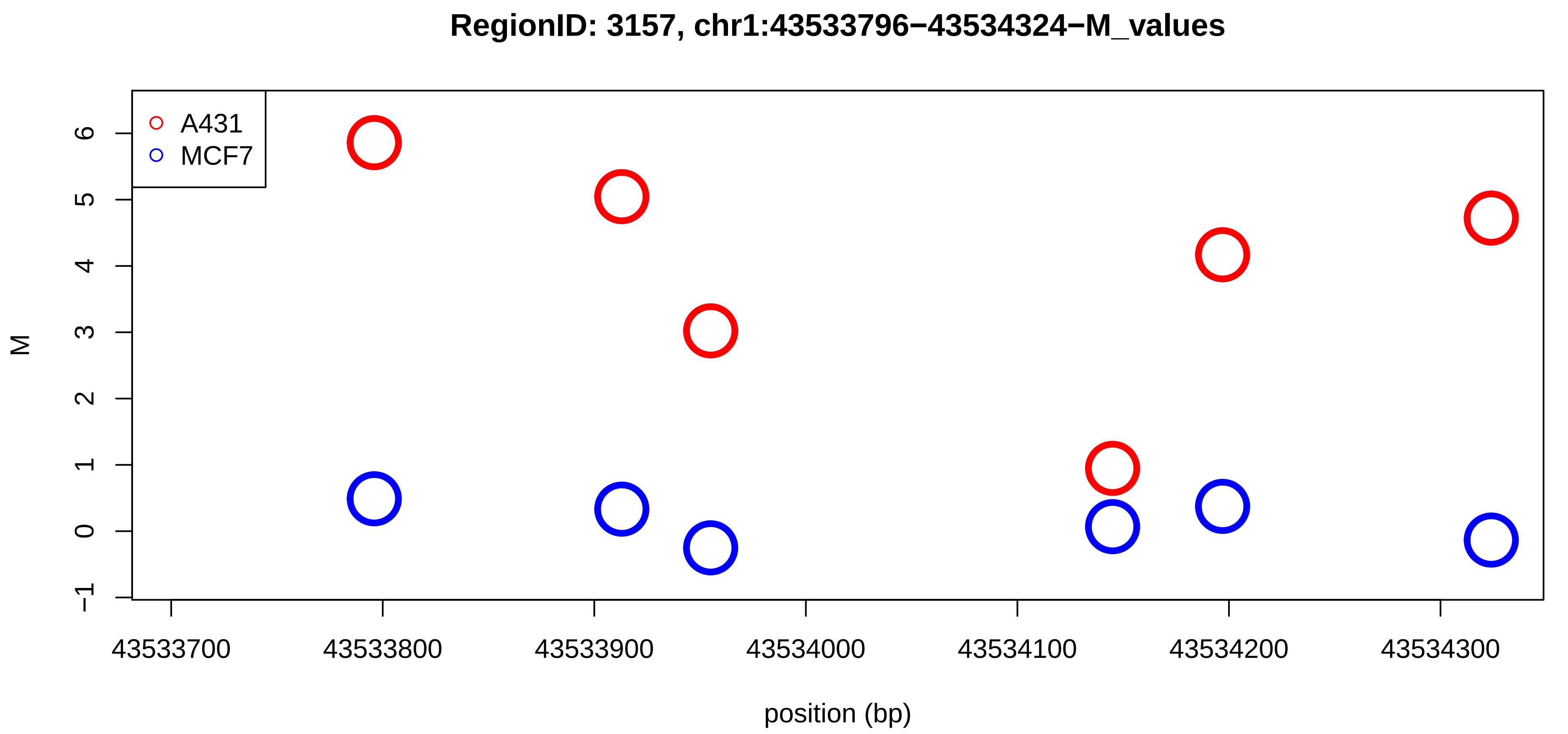

RegionID: 3157, chr1:43533796−43534324−M_values
A431
MCF7
M
−1
0
1
2
3
4
5
6
43533700
43533800
43533900
43534000
43534100
43534200
43534300
position (bp)

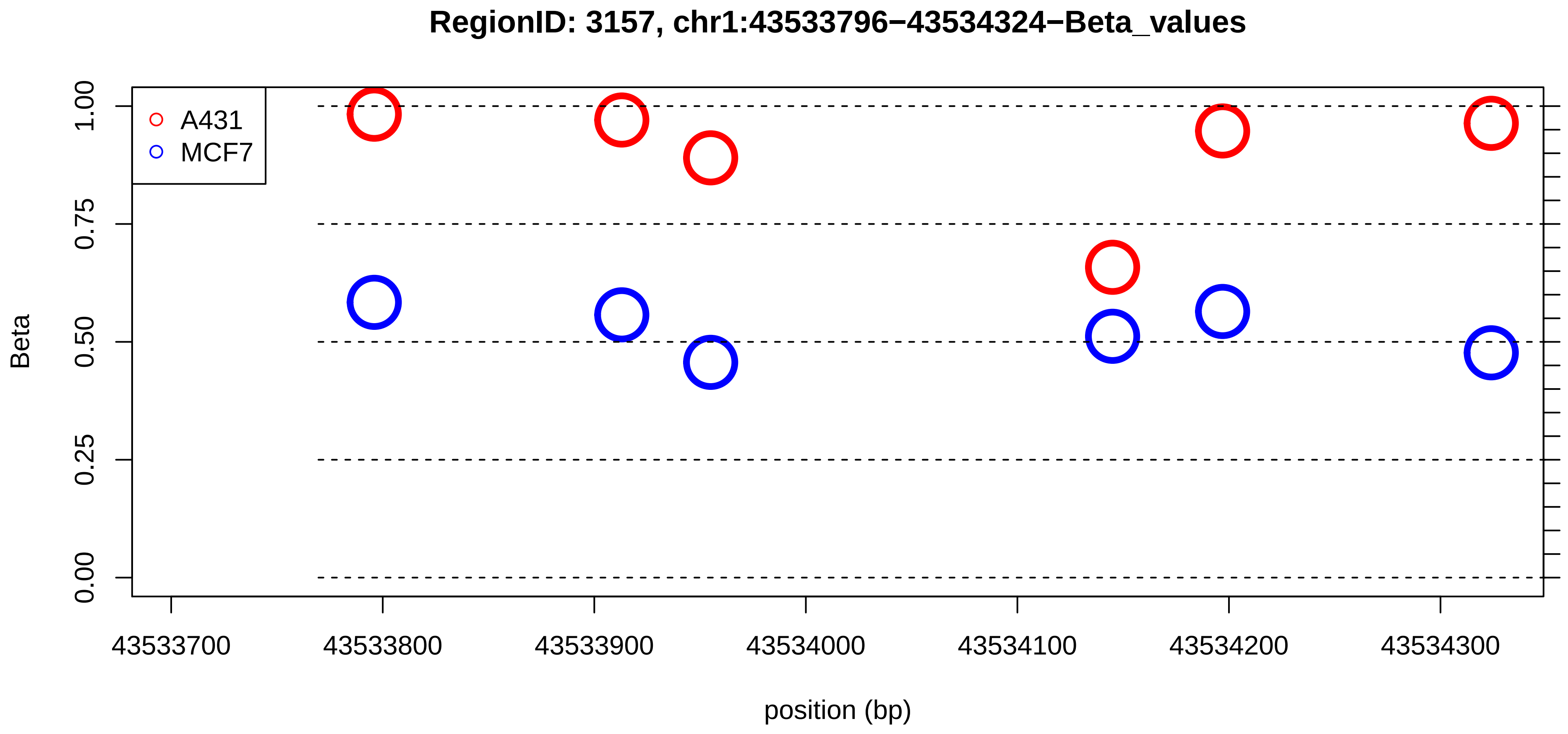

RegionID: 3157, chr1:43533796−43534324−Beta_values
A431
MCF7
Beta
0.00
0.25
0.50
0.75
1.00
43533700
43533800
43533900
43534000
43534100
43534200
43534300
position (bp)

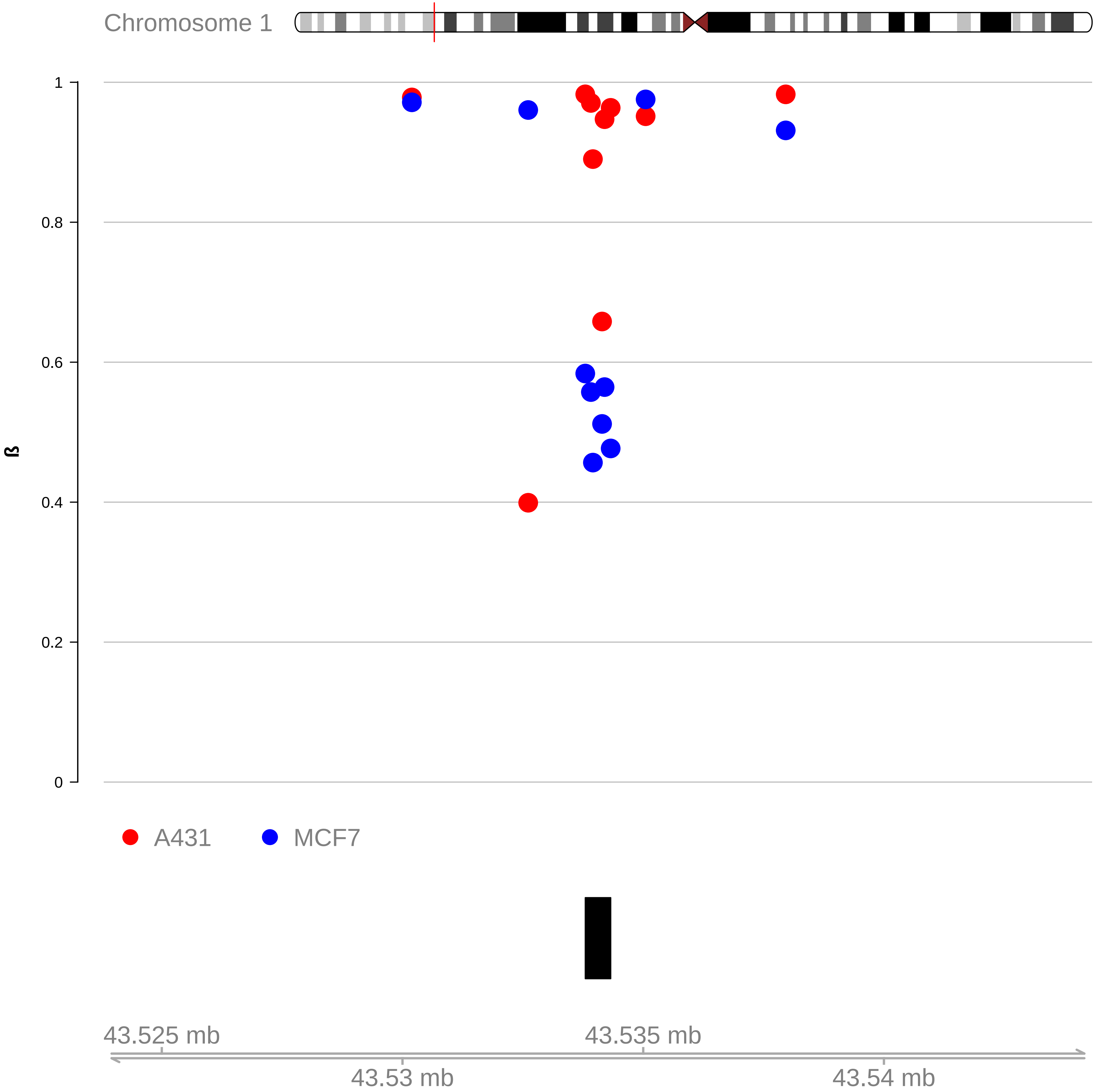

Chromosome 1
1
0.8
0.6
0.4
0.2
0
ß
A431
MCF7
43.525 mb
43.535 mb
43.53 mb
43.54 mb
